# A multicenter, open-label, single-arm phase I trial of neoadjuvant nivolumab monotherapy for resectable gastric cancer

**DOI:** 10.1007/s10120-022-01286-w

**Published:** 2022-03-07

**Authors:** Hirotaka Hasegawa, Kohei Shitara, Shuji Takiguchi, Noriaki Takiguchi, Seiji Ito, Mitsugu Kochi, Hidehito Horinouchi, Takahiro Kinoshita, Takaki Yoshikawa, Kei Muro, Hiroyoshi Nishikawa, Hideaki Suna, Yasuhiro Kodera

**Affiliations:** 1 Department of Gastroenterological Surgery, Nagoya University School of Medicine, 65 Tsurumai-cho, Showa-ku, Nagoya, 466-8560 Japan; 2 Department of Gastrointestinal Oncology, National Cancer Center Hospital East, Kashiwa, Japan; 3 Department of Gastroenterological Surgery, Graduate School of Medical Sciences, Nagoya City University, Nagoya, Japan; 4 Department of Surgery, Tsuchiura Kyodo General Hospital, Tsuchiura, Japan; 5 Department of Gastroenterological Surgery, Aichi Cancer Center Hospital, Nagoya, Japan; 6 Department of Digestive Surgery, Nihon University Itabashi Hospital, Tokyo, Japan; 7 Department of Thoracic Oncology, National Cancer Center Hospital, Tokyo, Japan; 8 Department of Gastric Surgery, National Cancer Center Hospital East, Kashiwa, Japan; 9 Department of Gastric Surgery, National Cancer Center Hospital, Tokyo, Japan; 10 Department of Clinical Oncology, Aichi Cancer Center Hospital, Nagoya, Japan; 11 Division of Cancer Immunology, Research Institute/Exploratory Oncology Research and Clinical Trial Center, National Cancer Center, Tokyo, Japan; 12 Department of Immunology, Nagoya University Graduate School of Medicine, Nagoya, Japan; 13 Clinical Development Planning Division, Ono Pharmaceutical Co., Ltd., Osaka, Japan

**Keywords:** Neoadjuvant therapy, Gastric cancer, Nivolumab, Biomarker, PD-L1

## Abstract

**Background:**

Nivolumab monotherapy has demonstrated superior efficacy in advanced unresectable gastric cancer (GC), but its impact on resectable GC remains unknown. This phase I study aimed to evaluate safety, feasibility, and potential biomarkers of neoadjuvant nivolumab monotherapy in resectable GC.

**Methods:**

Untreated, resectable, cT2 or more advanced gastric adenocarcinomas with clinical stage I, II, or III were treated with two doses of nivolumab before gastrectomy. Patients were excluded if their tumors may be applicable to neoadjuvant chemotherapy. The primary endpoint was the incidence of adverse event (AE) categories of special interest.

**Results:**

All of the 31 enrolled patients completed 2 doses of nivolumab monotherapy. While 30 (97%) patients underwent surgery with curative intent, 1 patient discontinued before the planned surgical intervention because of a newly emerging liver metastasis. Seven patients (23%) had nivolumab treatment-related AEs, and one patient had a treatment-related AE of grade 3–4. The incidences of treatment-related AE categories of special interest ranged from 0 to 6%. Notable surgical complications included two cases of grade 3 anastomotic leakage and two cases of pancreatic fistula. The major pathologic response (MPR) assessed by the independent pathology review committee was achieved in five (16%) patients, of which one patient had a pathologic complete response. The MPR was mostly observed in patients with positive PD-L1 expression, high microsatellite instability, and/or high tumor mutation burden.

**Conclusions:**

Neoadjuvant nivolumab monotherapy is feasible with an acceptable safety profile and induces a MPR in certain patients with resectable GC. (Registration: clinicaltrials.jp, JapicCTI-183895).

**Supplementary Information:**

The online version contains supplementary material available at 10.1007/s10120-022-01286-w.

## Introduction

Gastric cancer (GC) is one of the most common cancers in the world, with more than 1 million new incidences and approximately 770,000 deaths in 2020 [1]. Perioperative chemotherapy with triplet regimens in addition to surgery is the standard of care in the West [2–4]. Phase III evidence in this regard is yet to be generated in the Eastern hemisphere [5], where gastrectomy with lymphadenectomy followed by postoperative chemotherapy remains the standard [6, 7]. Nevertheless, Asian investigators have been keen to explore neoadjuvant chemotherapy, which has potential benefits in terms of early exposure of potential micrometastases to cytotoxic agents in addition to the likelihood of tumor shrinkage. These attempts have shown safety with a high compliance to the preoperative treatment in addition to down-staging and a high R0 resection rate in the neoadjuvant treatment arm [5].

Nivolumab is an immune checkpoint inhibitor of the programmed cell death 1 receptor (PD-1). Nivolumab monotherapy has demonstrated superior efficacy compared with placebo or standard chemotherapy in multiple types of cancer, including advanced GC, non-small-cell lung cancer (NSCLC), renal-cell carcinoma, and squamous-cell carcinoma of the head and neck [8–12]. However, although the efficacy of preoperative cytotoxic agents has been well documented in GC, the clinical and molecular effects of preoperative nivolumab monotherapy on GC tissue remain virtually unknown and are of considerable interest. Although the objective response rate of nivolumab monotherapy in heavily pretreated patients reaches 11.9% [13], the response to immunotherapy may not be negligible in relatively early stage GC; notably, nivolumab monotherapy demonstrated a pathologic response rate of up to 45% in resectable NSCLC [14, 15]. In addition, the establishment of optimal biomarkers can justify the delivery of this relatively less toxic treatment for selected patients.

Accordingly, we conducted this phase I study to assess the safety and feasibility of neoadjuvant nivolumab monotherapy in patients with resectable GC. The correlations between conventional biomarkers and the clinical response were also evaluated.

## Methods

### Study design

This study is a part of the ONO-4538-67 study (JapicCTI-183895), which is a multicenter, open-label, single-arm phase I trial of neoadjuvant nivolumab monotherapy for resectable malignancies of GC or NSCLC in Japan. Patients with resectable GC received 2 doses of intravenous nivolumab at a dose of 240 mg/body every 2 weeks, which is the same as the previous trial for NSCLC [14]. Gastrectomy with curative intent was scheduled at least 14 days after the last nivolumab dose. Thus, the duration of neoadjuvant therapy, including a 2-week interval before surgery, was designed to be as short as 4 weeks, given that the response rate of nivolumab monotherapy in heavily pretreated GC had been modest compared with optimal combinations of cytotoxic agents currently used in the neoadjuvant setting.

The primary endpoint was the incidence of adverse event (AE) categories of special interest, including endocrinopathies, gastrointestinal toxicities, hepatotoxicities, pulmonary toxicities, renal toxicities, dermatologic toxicities, and infusion reactions. The secondary endpoints were any AEs and efficacy, which included major pathologic response (MPR) rate, pathologic complete response (pCR) rate, shrinkage of the primary tumor assessed by endoscopy, and R0 resection rate. The other key endpoints were immunologic, genomic, and pathological correlations of responses in blood and tumor samples acquired by pretreatment endoscopic biopsy and surgery.

The study protocol was approved by the institutional review board or independent ethic committee at each study site. This study follows the Good Clinical Practice guidelines of International Council for Harmonisation. All patients provided a written informed consent in accordance with the Declaration of Helsinki.

### Patients

Patients were eligible for enrolment if they had untreated, resectable, cT2 or more advanced, histopathologically confirmed gastric adenocarcinoma with clinical stage I, II, or III, as indicated in the TNM Classification of Malignant Tumours (8th edition) of the Union for International Cancer Control [16]; were 20 years old or older; had Eastern Cooperative Oncology Group performance status (ECOG PS) of 0 or 1; had adequate organ function. Given the modest response rate of nivolumab monotherapy observed in heavily pretreated GC, patients were excluded if their tumor may be applicable to neoadjuvant chemotherapy, such as those with bulky lymph node (two or more adjacent lymph nodes of 1.5 cm diameter or a 3 cm diameter cluster of lymph nodes along the celiac, splenic, common hepatic, or proper hepatic arteries or the superior mesenteric vein) detected by contrast-enhanced abdominal computed tomography and linitis plastica-type GC [5, 17]. Cancer of the gastric remnant was also excluded. Other key exclusion criteria were locally advanced unresectable or metastatic tumors, severe malnutrition, and active autoimmune disorders. Only patients with an ECOG PS of 0 or 1, without major relevant clinical findings that may increase the risk of surgery to an unacceptable level, without disease progression in the form of distant metastasis, and with expected R0 resection were indicated for subsequent surgery. The administration of immunosuppressant, corticosteroids equivalent to > 10 mg/day prednisone, and other anticancer therapy were prohibited during the study period.

### Assessments

Patient characteristics, including laboratory values, were assessed upon the enrolment, periodically during the treatment and perioperative periods, and at 30 days after the surgery or 60 days after the last nivolumab dose, whichever was later.

We assessed the AEs occurring from the first nivolumab dose until 30 days after the surgery or 60 days after the last nivolumab dose, whichever was later, or until the time of study discontinuation. Intraoperative and postoperative complications were assessed until 30 days after surgery. Each AE was graded in accordance with the National Cancer Institute Common Terminology Criteria for Adverse Events version 4.0.

Tumors were assessed by computed tomography and magnetic resonance imaging of the chest, abdomen, and pelvis and by upper gastrointestinal endoscopy at the enrolment and just before surgery in accordance with the Response Evaluation Criteria in Solid Tumors (RECIST) guidelines (version 1.1). Responses in the primary tumor were assessed by upper gastrointestinal endoscopy and classified as follows: endoscopic complete response (eCR) if no tumor was observed; endoscopic partial response (ePR) if remarkable regression of the tumor was observed (≤ 2/3 in the diameter, ≤ 1/2 in the area, and ≤ 1/3 in the volume); endoscopic progressive disease (ePD) if the tumor was evidently enlarged; endoscopic stable disease (eSD) in other cases [18].

Surgical specimens, including primary gastric tumor and lymph nodes, were staged in accordance with the Japanese Classification of Gastric Carcinoma [16, 18]. The independent pathology review committee (IPRC) pathologically assessed the percentage of residual viable tumor that was identified on surgically resected specimens, which were stained routinely with hematoxylin and eosin, and tumors with no more than 10% viable tumor cells were considered to have had a MPR [19].

Tumor proportion score (TPS; the proportion of programmed cell death-ligand 1 (PD-L1)-positive cells among tumor cells) and combined positive score (CPS; the ratio of the sum of PD-L1-positive tumor cells, lymphocytes, and macrophages to the number of all tumor cells) were centrally assessed using archival tumor tissues with the PD-L1 IHC 28–8 pharmDx kit (Agilent Technologies Inc. Santa Clara, CA, USA). Tumor mutation burden (TMB) and microsatellite instability (MSI) were also assessed at the central laboratory using FoundationOne (Foundation Medicine Inc, Cambridge, MA, USA) and the MSI Analysis System (Promega Corp, Madison, WI, USA), respectively. Tumors with ≥ 10 mutations/Mb and with < 10 mutations/Mb were defined as TMB-High and TMB-Low, respectively.

### Statistics

The planned number of patients was 30. The average incidence of the AE categories of special interest was 10.8% in patients with unresectable advanced or metastatic GC that was refractory or intolerant to standard chemotherapy in the phase III ATTRACTION-2 study [8]. The incidence of AE categories of special interest was considered lower in this study than that in ATTRACTION-2 because only two doses were administered in this study. When the true incidence of the AE categories of special interest in this neoadjuvant nivolumab monotherapy was postulated as 3%, 5%, and 7%, at least 1 among the 30 patients might have experienced these AEs with a probability of 59.9%, 78.5%, and 88.6%, respectively. Thus, 30 patients may be sufficient to assess the AEs of special interest.

Safety was assessed in patients who received at least one dose of nivolumab. The efficacy was assessed in patients fulfilling the major eligible criteria. The pathologic response evaluable set constituted patients who underwent radical resection. The 95% confidence interval for MPR rates was estimated by the Clopper–Pearson method.

### Data availability

Qualified researchers may request Ono Pharmaceutical Co., Ltd. to disclose individual patient-level data from clinical studies through the following website: https://www.clinicalstudydatarequest.com/. For more information on the policy of Ono Pharmaceutical Co., Ltd. for the Disclosure of Clinical Study Data, please visit https://www.ono.co.jp/eng/rd/policy.html.

## Results

### Patients

Between November 2018 and December 2019, 31 patients were enrolled at 7 study sites. Most of the patients were male (68%) and had an ECOG PS score of 0 (97%) (Table 1). The primary tumor sites were the stomach in 97% of patients and the esophagogastric junction in 3% of patients (Online Resource 1). Lymph node metastasis was observed in 32% of patients, and most patients (68%) were clinically confined to stages I or II. Table 1 and Online Resource 1 summarize the other clinical characteristics of the tumors.

**Table 1:** Patient baseline characteristics

Characteristics	Nivolumab (*N* = 31)
Median age—years (range)	69 (44–84)
Sex—*n* (%)	
Male	21 (68)
Female	10 (32)
ECOG PS—*n* (%)	
0	30 (97)
1	1 (3)
T classification—*n* (%)	
T2	7 (23)
T3	22 (71)
T4a	2 (6)
T4b	0
N classification—*n* (%)	
N0	21 (68)
N1	7 (23)
N2	3 (10)
M classification—*n* (%)	
M0	31 (100)
M1	0
Clinical stage—*n* (%)	
I	7 (23)
IIA	0
IIB	14 (45)
III	10 (32)
PD-L1 TPS—*n* (%)	
< 1%	22 (71)
≥ 1 to < 10%	6 (19)
≥ 10%	3 (10)
PD-L1 CPS—*n* (%)	
< 1	11 (35)
≥ 1 to < 10	11 (35)
≥ 10	9 (29)
MSI status—*n* (%)	
MSI-high	7 (23)
MSI-low	4 (13)
MSS	20 (65)
TMB—*n* (%)	
High	9 (29)
Low	12 (39)
Missing^a^	10 (32)

*CPS* combined positive score, *ECOG PS* Eastern Cooperative Oncology Group performance status, *Mb* mega base pairs, *MSI* microsatellite instability, *MSS* microsatellite stable, *TMB* tumor mutation burden, *TPS* tumor proportion score

^a^Including those that were not evaluable and not determined

### Safety and feasibility

All of the 31 patients completed 2 doses of neoadjuvant nivolumab monotherapy; no AEs led to the discontinuation of nivolumab. AEs were observed in 21 (68%) patients, of which 9 (29%) were grade 3–4 AEs. Seven patients (23%) had nivolumab treatment-related AEs, and one patient (3%) had a treatment-related AE of grade 3–4, which was the grade 3 asymptomatic lipase increased. The most common AEs of special interest were alanine aminotransferase increased in four patients, aspartate aminotransferase increased in three, diarrhea in three, and rash in three (Table 2). Two patients had rash (grade 1 and 2) as nivolumab treatment-related AEs. Any AEs that have not been previously reported to be associated with nivolumab were not observed.

**Table 2:** AEs

*N* = 31	AEs		Treatment-related AEs	
	Any grade	Grade 3–4	Any grade	Grade 3–4
Any	21 (68)	9 (29)	7 (23)	1 (3)
AEs of special interest				
ALT increased	4 (13)	2 (6)	1 (3)	0
AST increased	3 (10)	1 (3)	0	0
Blood creatinine increased	1 (3)	0	0	0
Dermatitis	1 (3)	0	0	0
Diarrhea	3 (10)	0	1 (3)	0
Eczema	1 (3)	0	0	0
GGT increased	2 (6)	2 (6)	0	0
Hypothyroidism	1 (3)	0	1 (3)	0
Rash	3 (10)	0	2 (6)	0
Treatment-related AEs				
ALT increased	4 (13)	2 (6)	1 (3)	0
Cardiomyopathy	1 (3)	0	1 (3)	0
Diarrhea	3 (10)	0	1 (3)	0
Fatigue	1 (3)	0	1 (3)	0
Hypothyroidism	1 (3)	0	1 (3)	0
Lipase increased	1 (3)	1 (3)	1 (3)	1 (3)
Pancreatic fistula	2 (6)	0	1 (3)	0
Rash	3 (10)	0	2 (6)	0
Vomiting	1 (3)	0	1 (3)	0

We assessed the AEs that occurred from the first nivolumab dose until 30 days after the surgery or 60 days after the last nivolumab dose, whichever was later, or until the time of study discontinuation. The number (%) of patients is shown. Nivolumab treatment-related AEs are shown on the right

*AE* adverse event, *ALT* alanine aminotransferase, *AST* aspartate aminotransferase, *GGT* gamma-glutamyltransferase

Among the 30 patients who underwent surgery, 15 (50%) patients had intraoperative and postoperative complications of any grade (Online Resource 2). Notable surgical complications included two cases of grade 3 anastomotic leakage and two cases of pancreatic fistula. No intra-abdominal abscess was observed.

### Clinical efficacy

The proportions of patients with ePR and eSD were 13% and 84%, respectively (Online Resource 3). No patient with target lesions in accordance with the RECIST criteria was observed. Meanwhile, 30 (97%) patients underwent surgery with curative intent, and one (3%) patient who had a T4a tumor discontinued after completing the nivolumab treatment before the planned surgical intervention because of liver metastasis that emerged as a new lesion. One patient underwent surgery behind the schedule due to grade 2 rash. R0 resection was achieved in 27 patients (90% of the patients who underwent surgery with curative intent), whereas the remaining 3 patients had a macroscopic residual tumor (R2) resection due to peritoneal metastases that were undetected prior to enrolment.

### Pathological findings

Online Resource 4 depicts the tumor characteristics upon the enrolment (clinical assessments) and after surgery (pathological assessment). Pathological T2 or lower was observed in 15 patients (48%). The proportion of pathological stage 0/I patients was as high as 45%, whereas that of clinical stage I patients upon enrolment was 23%. On the other hand, no evident decrease in the proportion of ≥ stage III patients was observed. Although distant metastases had not been detected through imaging studies at the time of enrolment, one patient developed liver metastasis during the neoadjuvant immunotherapy, and three other patients had peritoneal metastasis that was recognized at surgery but not by imaging just before the surgery; no staging laparoscopy, when conducted, found peritoneal metastasis before enrolment.

**Fig. 1:**
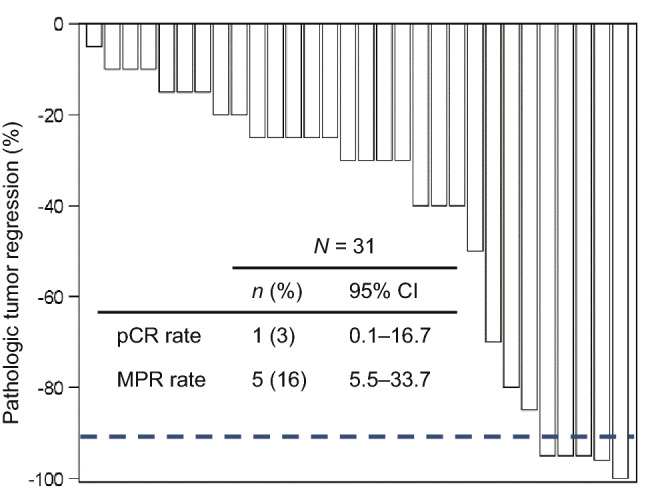
Pathologic response. The waterfall plot shows the tumor reduction ratio calculated from residual tumors in the resected specimens. The tumor reduction in the patient who did not undergo surgery was not evaluated. The dotted line indicates 90% reduction. *CI* confidence interval, *MPR* major pathologic response, *pCR* pathologic complete response

**Fig. 2:**
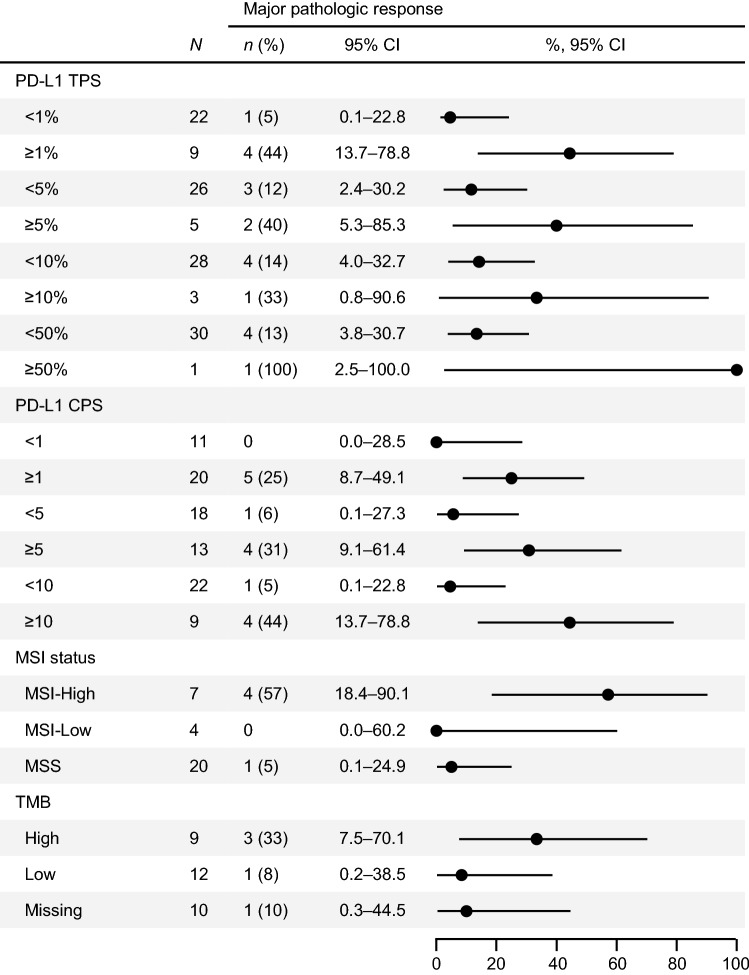
Major pathologic response rates. *CI* confidence interval, *CPS* combined positive score, *MSI* microsatellite instability, *MSS* microsatellite stable, *ND* not determined, *TMB* tumor mutational burden, *TPS* tumor proportion score

All of the 30 patients who underwent surgery provided specimens with diverse levels of tumor regression (Fig. 1). An IPRC-assessed MPR in the primary tumor was achieved in five (16%) patients, of which one had pCR. The patient who achieved pCR had a clinical stage I disease, whereas other cases of MPR were observed among patients with clinical stages IIB and III (Online Resource 5). Three out of five patients with MPR had ePR, and the other two, including one with pCR, had eSD. The majority of the patients with a MPR were those with ≥ 1% PD-L1 TPS (80%), ≥ 10 PD-L1 CPS (80%), MSI-High (80%), and TMB-High (60%) (Fig. 2). Among the patients without MPR, the proportions of patients with MSI-High and TMB-High were 12% and 20%, respectively (Online Resource 6).

Fibrosis without apparent tumor cells was observed in the surgically resected specimens of the patient who achieved pCR (Online Resource 7).

## Discussion

Neoadjuvant therapy with two doses of nivolumab in patients with GC was safe and had no new safety signal. Considering relatively high rate of disease progression after nivolumab for metastatic GC in the ATTRACTION-2 study [8], the number of the neoadjuvant treatment course had to be kept to minimal so as not to cause excessive delay in the surgical treatment. Because no prior information was available regarding efficacy of nivolumab monotherapy for resectable GC, we referred to the favorable results in the previous trial for NSCLC [14] and determined the duration of neoadjuvant treatment to be relatively short at 4 weeks. Nevertheless, two doses of nivolumab led to a MPR in 16% of patients who underwent surgery with curative intent. The R0 resection rate was favorable at 90%.

Meanwhile, more than 80% of the patients treated with neoadjuvant chemotherapy for GC reported treatment-related AEs [3, 20, 21]; the neoadjuvant nivolumab monotherapy in this study had a lower incidence of treatment-related AEs at 23%, which was comparable with the neoadjuvant nivolumab monotherapy for lung cancer (23%) [14]. The incidences of treatment-related AEs of special interest ranged from 0 to 6%, which were favorable compared with those observed during nivolumab monotherapy in the salvage line for advanced GC [8], although a direct comparison may be inappropriate given that only two doses of nivolumab were administered in this study.

Although one patient (3%) failed to undergo surgery due to a new lesion, the other patients (97%) were subsequently treated with surgery, in which the R0 resection rate was 90%. Five patients achieved a MPR, including one pCR, and the number of patients with pathologically stage I disease was twice the number of patients who had clinically stage I disease at the initial presentation, suggesting the promising efficacy of neoadjuvant nivolumab monotherapy. In the FLOT4 study, the representative neoadjuvant chemotherapy achieved a 94% gastrectomy rate; however, 2% of patients suffered from disease progression or died during the neoadjuvant chemotherapy [3]. Other studies evaluating neoadjuvant chemotherapy for GC also demonstrated comparable gastrectomy and disease progression rates [20, 21]. The MPR rate in neoadjuvant nivolumab monotherapy (16%) was also comparable with those (6%–32%) in patients with GC receiving neoadjuvant chemotherapy [4, 20–22]. However, such comparisons should be interpreted with caution, given that patients enrolled in the current study had GC at relatively early clinical stages. Although the planned treatment duration was designed to be relatively short at 4 weeks, at least one patient had a new lesion prior to surgery, and 3 other patients failed to undergo R0 resection due to macroscopic metastases that were inevident at the time of enrolment. We plan to seek for effect-predictive biomarkers in the forthcoming molecular analyses using clinical specimens collected before and after the nivolumab monotherapy.

Regarding several potential biomarkers that have already been evaluated, nivolumab and pembrolizumab monotherapies for pretreated or treatment-naïve GC have demonstrated relatively high response rates in patients with MSI [23–25]. A similar trend was observed in other tumors, including gastroesophageal cancer, colorectal cancer, and lung cancer [26–30]. Consistent with these reports, a high MPR (57%) was observed in MSI-High tumors after two doses of nivolumab monotherapy in the neoadjuvant setting compared with those (0–11%) observed in perioperative chemotherapy [31–33]. Given that cytotoxic agents remain the current standard perioperative therapy for resectable GC despite relatively poor efficacy for MSI-High tumors, this study may open the key for the future trial of neoadjuvant nivolumab monotherapy for resectable MSI-High GC. On the other hand, GC tumors with MSI-Low or microsatellite stable (MSS) responded well to perioperative chemotherapy [31–34], but poorly to the neoadjuvant nivolumab monotherapy. Mechanisms underlying the low response in MSI-Low and MSS remain to be addressed. One MSS tumor achieved a MPR, in which the PD-L1 TPS and CPS scores were 5% and 15, respectively, and the TMB status was low, suggesting that the relatively high PD-L1 expression level might have contributed to the response. However, the sample size was too small to assess the correlation between the PD-L1 expression levels and response to neoadjuvant nivolumab monotherapy. Pembrolizumab monotherapy at the salvage line for advanced GC demonstrated higher benefits in patients with PD-L1-positive than in patients with PD-L1-negative [35], whereas the ATTRACTION-2 study demonstrated the benefit of nivolumab monotherapy regardless of the PD-L1 expression levels although PD-L1 TPS was determined only in a limited number of patients [13]. The benefit of neoadjuvant nivolumab monotherapy on patients with lung cancer was obtained regardless of the PD-L1 expression levels [14]. The controversial issue regarding response to immunotherapy and PD-L1 expression status will be addressed in the future. Furthermore, the resected specimens obtained in this study were valuable in ascertaining the immune effector function of nivolumab within the GC microenvironment during the therapeutic time window. In the meantime, a combination of perioperative chemotherapy with immunotherapy may be promising, as shown in untreated, unresectable GC in CheckMate 649 and ATTRACTION-4 studies, and therefore has been evaluated for resectable GC in ongoing phase III clinical trials, including ATTRACTION-5 study for nivolumab, KEYNOTE-585 study for pembrolizumab, and MATTERHORN study for durvalumab.

The small number of patients limited the interpretation of the results, especially in the subpopulation analysis. Other limitations of this study include the absence of a comparator group, lack of ethnic diversity, and relatively early stages of tumors. Long-term follow-up is required to assess the advantages of neoadjuvant nivolumab monotherapy, given that MPR only serves as a surrogate of survival endpoints.

In conclusion, neoadjuvant nivolumab monotherapy is feasible with an acceptable safety profile, and it induces a MPR in certain patients with resectable GC despite the short duration. Further molecular analyses for the identification of predictive markers of the response are awaited to establish an optimal neoadjuvant therapy with nivolumab, either alone or in combination with cytotoxic agents.

## Supplementary Information

Below is the link to the electronic supplementary material.

Supplementary file1 (PDF 598 KB)
