# Supplementary material for: A multicenter, open-label, single-arm phase I trial of neoadjuvant nivolumab monotherapy for resectable gastric cancer
Source: Gastric Cancer. 2022 Mar 7;25(3):619–28. doi: 10.1007/s10120-022-01286-w (PMC9013329; doi:10.1007/s10120-022-01286-w)
Supplement: Supplementary file 1 — Supplementary file1 (PDF 598 KB) [file 10120_2022_1286_MOESM1_ESM.pdf]

Gastric Cancer

Electronic supplementary material

A multicenter, open-label, single-arm phase I trial of neoadjuvant nivolumab monotherapy for resectable gastric cancer

Hirotaka Hasegawa[1], Kohei Shitara[2], Shuji Takiguchi[3], Noriaki Takiguchi[4], Seiji Ito[5], Mitsugu Kochi[6], Hidehito Horinouchi[7], Takahiro Kinoshita[8], Takaki Yoshikawa[9], Kei Muro[10], Hiroyoshi Nishikawa[11,12], Hideaki Suna[13], and Yasuhiro Kodera[1]

[1]Department of Gastroenterological Surgery, Nagoya University School of Medicine, Japan; [2]Department of Gastrointestinal Oncology, National Cancer Center Hospital East, Japan; [3]Department of Gastroenterological Surgery, Graduate School of Medical Sciences, Nagoya City University, Japan; [4]Department of Surgery, Tsuchiura Kyodo General Hospital, Japan; [5]Department of Gastroenterological Surgery, Aichi Cancer Center Hospital, Japan; [6]Department of Digestive Surgery, Nihon University Itabashi Hospital, Japan; [7]Department of Thoracic Oncology, National Cancer Center Hospital. Japan; [8]Department of Gastric Surgery, National Cancer Center Hospital East, Japan; [9]Department of Gastric Surgery, National Cancer Center Hospital, Japan; [10]Department of Clinical Oncology, Aichi Cancer Center Hospital, Japan; [11]Division of Cancer Immunology, Research Institute/Exploratory Oncology Research and Clinical Trial Center, National Cancer Center, Japan; [12]Department of Immunology, Nagoya University Graduate School of Medicine, Japan; and [13]Clinical Development Planning Division, Ono Pharmaceutical Co., Ltd., Japan.

Corresponding author: Yasuhiro Kodera, Department of Gastroenterological Surgery, Nagoya University School of Medicine, 65 Tsurumai-cho, Showa-ku, Nagoya 466-8560, Japan

E-mail: ykodera@med.nagoya-u.ac.jp

Online Resource 1. Patient baseline characteristics

Characteristics	Nivolumab ($N = 31$)
Primary tumor site	
Esophagogastric junction	1 (3)
Gastric fundus	3 (10)
Gastric corpus	12 (39)
Pylorus and antrum	15 (48)
Macroscopic Borrmann classification	
Early gastric cancer	4 (13)
Type 1	1 (3)
Type 2	17 (55)
Type 3	9 (29)
Histological Lauren classification	
Intestinal	11 (35)
Diffuse	19 (61)
Mix	1 (3)

The number (%) of patients is shown.

Online Resource 2. Perioperative complications in patients undergoing radical radiation

$N = 30$	Any grade	Grade 3–4
Any	15 (50)	6 (20)
Splenic vein occlusion	1 (3)	0
Diarrhea	1 (3)	0
Dumping syndrome	2 (7)	0
Nausea	2 (7)	0
Pancreatic fistula	2 (7)*	0
Fistula of the small intestine	1 (3)	1 (3)
Pyrexia	3 (10)	0
Arterial injury	1 (3)	1 (3)
Failure to anastomose	1 (3)	1 (3)
Anastomotic leak	1 (3)	1 (3)
Procedural pain	10 (33)	0
ALT increased	3 (10)	2 (7)
AST increased	3 (10)	1 (3)
Blood creatine phosphokinase increased	1 (3)	0
GGT increased	2 (7)	2 (7)
Hyponatremia	1 (3)	0
Decreased appetite	1 (3)	1 (3)
Dizziness	1 (3)	0
Headache	1 (3)	0
Insomnia	3 (10)	0
Atelectasis	1 (3)	0
Pneumonia aspiration	1 (3)	0

Perioperative complications were AEs that occurred during the surgery and the following 30 days and were attributed to surgical procedures. The number (%) of patients is shown.
*One event was considered related to nivolumab.

Online Resource 3. Endoscopic evaluation of tumor response

	Nivolumab (N = 31)	
	n (%)	95% CI
eCR	0	0.0–11.2
ePR	4 (13)	3.6–29.8
eSD	26 (84)	66.3–94.5
ePD	0	ND
NE	1 (3)	ND

Data are the number (%) of patients and 95% confidence interval (95% CI) of the patient proportions. 95% CI was estimated by the Clopper–Pearson method.
eCR, endoscopic complete response; ePD, endoscopic progressive disease; ePR, endoscopic partial response; eSD, endoscopic stable disease; ND, not determined; NE, not evaluable.

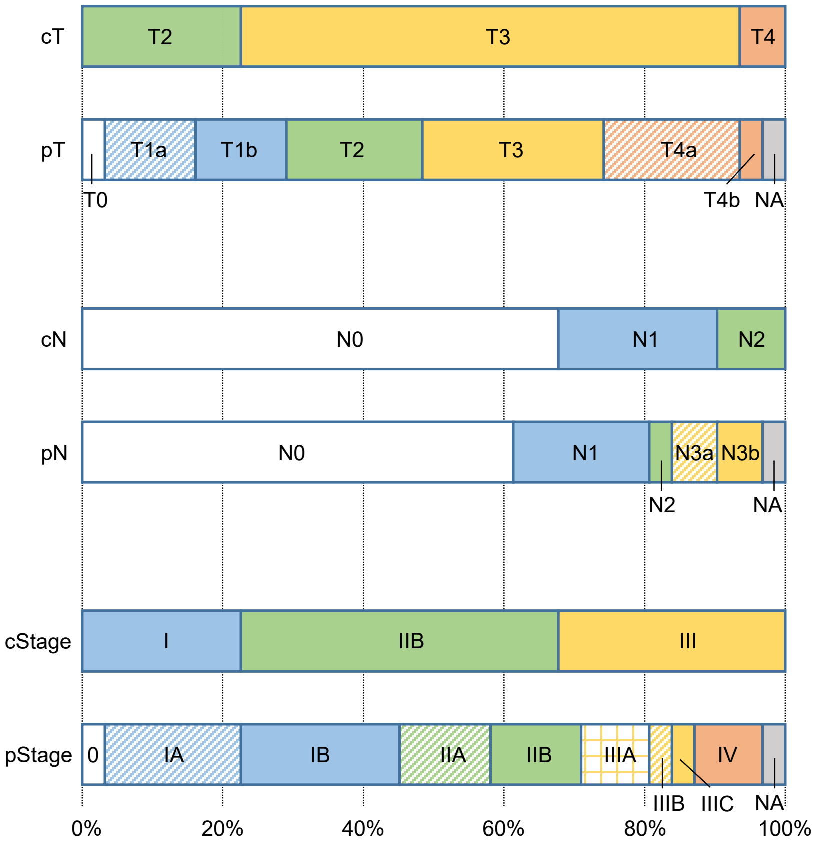

Online Resource 4. Tumor classification
Clinical T, N, and stage at the diagnosis and pathological T, N, and stages after the surgery are depicted. $N = 31$. NA, not applicable due to incapacity of patient to undergo surgery.

Online Resource 5. Characteristics of tumors and biomarker status in patients with MPR

	Patient 1	Patient 2	Patient 3	Patient 4	Patient 5
Response	pCR	MPR	MPR	MPR	MPR
Endoscopic Response	eSD	ePR	ePR	ePR	eSD
Resection	R0	R0	R0	R0	R0
cT	cT2	cT3	cT3	cT3	cT3
pT	pT0	pT1a	pT1a	pT1a	pT4a
cN	cN0	cN0	cN1	cN1	cN0
pN	pN0	pN0	pN0	pN0	pN1
cStage	cI	cIIB	cIII	cIII	cIIB
pStage	p0	pIA	pIA	pIA	pIIIA
PD-L1 TPS	0%	70%	3%	5%	2%
PD-L1 CPS	1	80	20	15	10
MSI status	High	High	High	MSS	High
TMB[a]	Missing	High	High	Low	High

[a] The number of mutations per mega-bases is shown in TMB.

c, clinical; CPS, combined positive score; CR, complete response; ePR, endoscopic partial response; eSD, endoscopic stable disease; MPR, major pathologic response; MSI, microsatellite instability; MSS, microsatellite stable; p, pathological; TMB, tumor mutation burden; TPS, tumor proportion score.

Online Resource 6. Characteristics of patients with and without MPR

	patients with MPR	Patients without MPR	Patients with MPR / all patients					
			MSI-High	MSI-Low	MSS	TMB-High	TMB-Low	TMB-missing[a]
	N = 5	*N* = 25	*N* = 4/7	*N* = 0/4	*N* = 1/19	*N* = 3/8	*N* = 1/12	*N* = 1/10
Resection								
R0	5 (100)	22 (88)	4/7	0/3	1/17	3/8	1/10	1/9
R2	0	3 (12)	0/0	0/1	0/2	0/0	0/2	0/1
PD-L1 TPS								
<1%	1 (20)	21 (84)	1/3	0/3	0/16	0/5	0/9	1/8
≥1% to <10%	3 (60)	3 (12)	2/2	0/1	1/3	2/2	1/3	0/1
≥10%	1 (20)	1 (4)	1/2	0/0	0/0	1/1	0/0	0/1
PD-L1 CPS								
<1	0	11 (44)	0/2	0/3	0/6	0/3	0/3	0/5
≥1 to <10	1 (20)	10 (40)	1/1	0/1	0/9	0/1	0/6	1/4
≥10	4 (80)	4 (16)	3/4	0/0	1/4	3/4	1/3	0/1
MSI status								
MSI-High	4 (80)	3 (12)	NA	NA	NA	3/5	0/0	1/2
MSI-Low	0	4 (16)	NA	NA	NA	0/0	0/1	0/3
MSS	1 (20)	18 (72)	NA	NA	NA	0/3	1/11	0/5
TMB								
High	3 (60)	5 (20)	3/5	0/0	0/3	NA	NA	NA
Low	1 (20)	11 (44)	0/0	0/1	1/11	NA	NA	NA
Missing[a]	1 (20)	9 (36)	1/2	0/3	0/5	NA	NA	NA

The number (%) of patients is shown.

CPS; combined positive score; ECOG PS, Eastern Cooperative Oncology Group performance status; Mb, mega base pairs; MSI, microsatellite instability; MSS, microsatellite stable; NA, not applicable; TMB, tumor mutation burden; TPS, tumor proportion score.

[a] including those that were not evaluable and not determined.

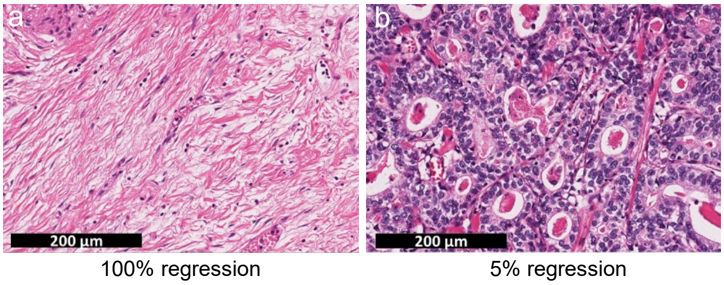

Online Resource 7. Patterns of pathologic response.
Complete pathologic response (a, 100% regression) and residual disease (b, 5% regression) after neoadjuvant nivolumab monotherapy in two resected specimens.
